# MJMS Performance Reports 2014–2018: The Unique Profile of the Top Submitting Authors

**DOI:** 10.21315/mjms2020.27.2.18

**Published:** 2020-04-30

**Authors:** Irfan Mohamad

**Affiliations:** Department of Otorhinolaryngology - Head & Neck Surgery, School of Medical Sciences, Universiti Sains Malaysia, Kelantan, Malaysia

Dear Editor,

I read with great interest the recent issue’s Editorial about the *Malaysian Journal of Medical Sciences* (MJMS)’ performance status in 2018 (1). It was really an eye-catching commentary that noted manuscripts received from the Republic of Iran constituted almost 22.8% (82 out of 359) of the total submissions of 2018. Similar statistics were seen in the previous year of 2017, placing Iranian authors second only to Malaysian authors (2). For the record, the Republic of Iran has been among the top three countries, along with India, with the highest submission of manuscripts for five consecutive years from 2014–2018 (3–5). In addition, a pattern of increased submissions from Iranian authors as a percentage of total submissions was distinctly seen in the three most recent years: 13.6% in 2016; 16.4% in 2017 and, as noted above, 22.8% in 2018 (1–3).

However, the question of ‘Why Iran?’ that I sought to answer was not found in the Editorial. Does MJMS data include the percentage of accepted manuscripts according to the country of origin? If the answer is ‘Yes’ and if Iranian papers were published frequently in MJMS, then the postulation that Iranian authors talked about their publication acceptance with others is likely to be true. Another question to scrutinise: Were the submitting authors from the same institution and, thus, more likely to have shared within their contacts? Maybe the database can enlighten us.

The increasing trend of submissions amongst Iranian authors is indeed a unique feature. However, if MJMS could identify why this unique feature existed, this would be very interesting. Papers received from the Middle East in 2014 included Iran (42 manuscripts), followed by Turkey (five manuscripts), Saudi Arabia (five manuscripts) and Iraq (one manuscript) (5). As the numbers indicate, no other neighbouring country in the region came close to the Republic of Iran’s total number of submissions. This may point to factors that encourage Iranian authors to send manuscripts to MJMS; on the other hand, MJMS may be drawn to the work of Iranian authors. Another possibility exists—if the acceptance rate of Iran papers is high—that reflects the suitability of the study areas of Iranian research in relation to the scope of MJMS.

As elegantly stated on the journal’s introductory web page, MJMS accepts high-quality papers especially from low- and middle-income countries (6). Iran (upper-middle income) and India (lower-middle income) represent the best examples of how MJMS lives up to its ideals.

## References

[1] Nour Azimah Z, Abdullah JM (2019). Malaysian Journal of Medical Sciences’ performance status in 2018. Malays J Med Sci.

[2] Nour Azimah Z, Abdullah JM (2018). Malaysian Journal of Medical Sciences’ performance status in 2017 and the challenges. Malays J Med Sci.

[3] Nour Azimah Z, Jafri Malin A (2017). Malaysian Journal of Medical Sciences’ performance status in 2016. Malays J Med Sci.

[4] Zulkapli NA, Sobi S, Mohd Zubaidi NA, Abdullah JM (2016). Malaysian Journal of Medical Sciences’ publishing report (2014–2015). Malays J Med Sci.

[5] Nur Farahin G, Norfatiha CA, Abdullah JM (2015). Malaysian Journal of Medical Sciences: a step forward towards an international journal. Malays J Med Sci.

[6] The World Bank World bank country and lending groups [Internet]; (Year of publication unknown).

